# Transglycosylation by a chitinase from *Enterobacter cloacae* subsp. *cloacae* generates longer chitin oligosaccharides

**DOI:** 10.1038/s41598-017-05140-3

**Published:** 2017-07-11

**Authors:** Mohan Krishna Mallakuntla, Papa Rao Vaikuntapu, Bhoopal Bhuvanachandra, Subha Narayan Das, Appa Rao Podile

**Affiliations:** Department of Plant Sciences, School of Life Sciences, University of Hyderabad, Gachibowli, Hyderabad, 50046 Telangana India

## Abstract

Humans have exploited natural resources for a variety of applications. Chitin and its derivative chitin oligosaccharides (CHOS) have potential biomedical and agricultural applications. Availability of CHOS with the desired length has been a major limitation in the optimum use of such natural resources. Here, we report a single domain hyper-transglycosylating chitinase, which generates longer CHOS, from *Enterobacter cloacae* subsp. *cloacae* 13047 (*Ec*Chi1). *Ec*Chi1 was optimally active at pH 5.0 and 40 °C with a K_m_ of 15.2 mg ml^−1^, and *k*_cat_/K_m_ of 0.011× 10^2^ mg^−1^ ml min^−1^ on colloidal chitin. The profile of the hydrolytic products, major product being chitobiose, released from CHOS indicated that *Ec*Chi1 was an endo-acting enzyme. Transglycosylation (TG) by *Ec*Chi1 on trimeric to hexameric CHOS resulted in the formation of longer CHOS for a prolonged duration. *Ec*Chi1 showed both chitobiase and TG activities, in addition to hydrolytic activity. The TG by *Ec*Chi1 was dependent, to some extent, on the length of the CHOS substrate and concentration of the enzyme. Homology modeling and docking with CHOS suggested that *Ec*Chi1 has a deep substrate-binding groove lined with aromatic amino acids, which is a characteristic feature of a processive enzyme.

## Introduction

Chitin [(C_8_H_13_O_5_N) _n»1_], is a linear homopolymer of *N*-acetyl glucosamine (GlcNAc) units linked through *β* (1 → 4) glycosidic bonds. It is an abundant renewable natural resource next to cellulose in the biosphere. Chitin is a primary structural component of the fungal cell wall, insects, molluscs, squid, internal shells of cephalopods and the exoskeletons of arthropod, exists in three biological forms i.e. *α*-chitin, *β*-chitin and *γ*-chitin. The presence of acetamide group (NH–CO–CH_3_) allows increased hydrogen bonding between adjacent polymers, giving increased strength of chitin – polymer matrix. Chitin oligosaccharides (CHOS) generated from polymeric chitin with specific composition and length have potential applications. The remarkable properties of CHOS i.e. bio-degradability, bio-compatibility, and non-toxicity suit wider industrial application^1, 2^. Production of CHOS by chemical methods has been challenging due to disadvantages like non-specific random hydrolysis, and difficulty to remove the acidity of the CHOS oligomers^3^. Chitinases could be possible alternatives for production of long chain CHOS, especially for biological applications.

Chitinases (EC 3.2.1.14) hydrolyze chitin to chitin monomer (DP1; Degree of polymerization 1), chitin dimer (DP2) and CHOS of shorter length. Chitinases, classified into two glycoside hydrolase (GH) families i.e. GH18 and GH19 could be exochitinases that act terminally and endochitinases that cleave randomly at internal sites of the chitin, eventually producing a variety of low molecular mass or short length CHOS^4^. Chitinases that act on crystalline polysaccharides need to associate and disrupt the polymer from packing and also direct the travelling of single polymer chain into the catalytic center through a catalytic groove, a mechanism called processivity^5, 6^. The aromatic amino acids lining the substrate-binding site, facilitate processivity by functioning as a flexible and hydrophobic sheet and allow the polymer chain to slide^7, 8^.

A few of the GH18 chitinases exhibit an uncommon transglycosylation (TG), in addition to chitin hydrolysis, by introducing new glycosidic bonds between donor and acceptor saccharides^9^. The GH18 chitinases gained special interest due to potential applications for the enzymatic production of CHOS by TG from chitin^10^. Bacterial chitinases like chitinase-D from *Serratia proteamaculans* (*Sp*ChiD) exhibited hyper-TG with chitotriose to chitohexaose (DP3–6) substrates generating products longer than chitohexaose (DP7–13)^11^. Similarly, chitinase A from *Stenotrophomonas maltophilia* ^12^ and ChiCW from *Bacillus cereus* 28–9 synthesized chitohexaose (DP6) from chitotetraose (DP4)^13^. A close relationship between bacterial chitinases and their pathogenicity may exist^14, 15^, and the molecular targets of chitinases in chitinous or non-chitinous hosts remain unclear. Hence, detailed understanding of chitinases at the biochemical or functional level may help in understanding their role in pathogenicity.

*Enterobacter cloacae* subsp. *cloacae* (*Ec*), a nosocomial pathogen, was originally isolated from the human cerebrospinal fluid by Edwin Oakes Jordan in 1890^16^. Analysis of the genome sequence of *E. cloacae* subsp. *cloacae* revealed that four chitinases and two *N*-acetyl-glucosaminidases could be involved in chitin degradation. In addition, *E. cloacae* genome has genes that code for one CBM-33 lytic polysaccharide monooxygenase (LPMO-GenBank: ADF60226.1) and one polysaccharide deacetylase (PDA-GenBank: ADF62202.1), which may have a crucial role in depolymerization of chitin. Of the four chitinases, two belong to family GH18 (*Ec*Chi1-GenBank: ADF62010.1 and *Ec*Chi2 (GenBank: ADF62328.1) and other two belong to GH19 (*Ec*Chi3-GenBank: ADF62326.1 and *Ec*Chi4-GenBank: ADF61237.1). Here, the *Ec*Chi1 with only the catalytic GH18 domain and no auxiliary domains displayed an unprecedented hyper-TG. We show that *Ec*Chi1 has structural features of a hyper-TG chitinase similar to *Sp*ChiD of *Serratia proteamaculans*.

## Results

### Cloning, heterologous expression, purification and dot blot assay

Full-length DNA sequence coding for *Ec*Chi1 consisted of 1257 nucleotides and an ORF of 418 amino acid residues with a predicted isoelectric point of 6.15. A 1.2-kb segment of *Ec*Chi1 gene was amplified with specific primers using gDNA of *E. cloacae* subsp. *cloacae* as a template, cloned into pET-28a (+), and transformed *E. coli* Rosetta-gami for expression. Extracellular protein was isolated from the induced *E. coli* Rosetta-gami culture pellet and purified *Ec*Chi1 through Ni-NTA affinity chromatography. The molecular mass of *Ec*Chi1 (42.5 kDa) calculated from amino acid sequence without the signal peptide was in agreement with experimentally observed molecular weight as obtained by 12% SDS-PAGE (Supplementary Fig. S1A). Dot - blot assay revealed that, *Ec*Chi1 exhibited clear zone on water-soluble glycol chitin containing gel confirming the chitinase activity (Supplementary Fig. S1B).

### Steady state kinetics of *Ec*Chi1 on colloidal chitin and chitobiose

**Figure 1:**
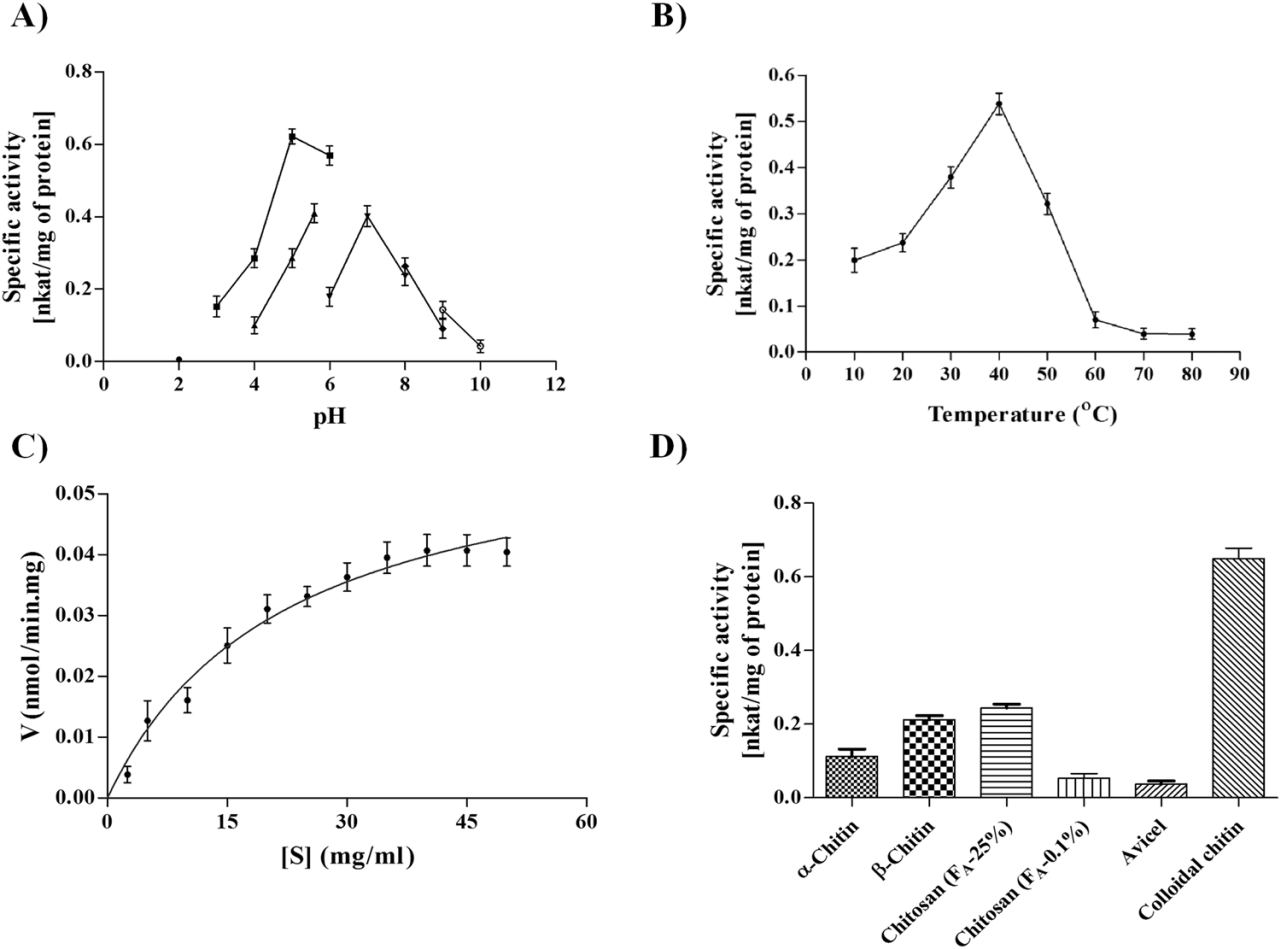
pH, temperature optima, kinetic parameters and activity on different insoluble substrates of *Ec*Chi1. The pH and temperature optima were determined by incubating 30 mg/ml colloidal chitin and 30 μg of *Ec*Chi1 for 1 h at 40 °C in various buffers at different pH values, as follows: (**A**) 50 mM glycine-HCl (pH 2.0, ▼), 50 mM sodium citrate (pH 3.0–6.0, ■), 50 mM sodium acetate (pH 4.0–6.0, ▲), 50 mM sodium phosphate (pH 6.0–8.00, ●), 50 mM Tris-HCl (pH 8.0–9.0, ♦) and 50 mM glycine-NaOH (pH 9.0–10.0, ○) and at 10 °C to 80 °C (**B**), Different concentrations of colloidal chitin (2.5–60 mg/ml) and 30 μg of *Ec*Chi1 in 50 mM sodium citrate buffer, pH 5.0. The average of the triplicate data was fitted to the Michaelis-Menten equation by a nonlinear regression function of graphpad prism version 6.0 (**C**), The reaction mixture containing 30 μg *Ec*Chi1 and 1 mg ml^−1^ of one of the polymeric substrates, was incubated at pH 5.0 and 40 °C for 1 h. The reaction mixture was centrifuged, and products were quantified using a reducing group assay (**D**). The error bars represent the standard deviations from three individual experiments.

The *Ec*Chi1 was active in mildly acidic to a neutral range of pH 5.0 to 7.0 with the optimum activity in 50 mM sodium citrate pH 5.0 (Fig. 1A). However, the activity decreased sharply below pH 5.0 and above 7.0, with diminished or no activity below pH 4.0 and above 9.0. *Ec*Chi1 was optimally active at 40 °C (Fig. 1B) and retained 60% activity at 50 °C, that was decreased with increase of temp up to 80 °C, while 70% activity was retained at 30 °C. The effect of metal ions on the activity of *Ec*Chi1 was also evaluated using colloidal chitin as substrate. The activity of *Ec*Chi1 in presence of Cu^+2^ was similar to control but was partially inhibited in the presence of urea, Ca^+2^, Mg^+2^, Zn^+2^, Mn^+2^, EDTA, and Fe^+2^. The presence of Hg^+2^ and SDS inhibited the activity by 80% and 90%, respectively. There was no increase in the activity of *Ec*Chi1 in the presence of test cations. The kinetic parameters of *Ec*Chi1 were determined with colloidal chitin and chitobiose as the substrate. The velocity measurements fitted to Michaelis-Menten kinetics revealed that the K_m_, *k*_cat_, and *k*_cat_/K_m_ values were 15.2 mg ml^−1^, 0.16 × 10^2 ^ min^−1^ and 0.011 × 10^2^ mg^−1^ ml min^−1^ for colloidal chitin (Fig. 1C) and 213.2 μM, 1.41 min^−1^ and 0.6 × 10^−2^ for chitobiose (Supplementary Fig. S2).

### Activity of *Ec*Chi1 on different insoluble substrates

*Ec*Chi1 exhibited maximum activity on colloidal chitin followed by chitosan, *β*-chitin, *α*-chitin and avicel. The activity of *Ec*Chi1 was 41% on chitosan (F_A_-25%), 35% on *β*-chitin, 17% on *α*-chitin, 8% on chitosan (F_A_-0.1%), and 5.7% on avicel.

### Binding study with soluble substrates

**Figure 2:**
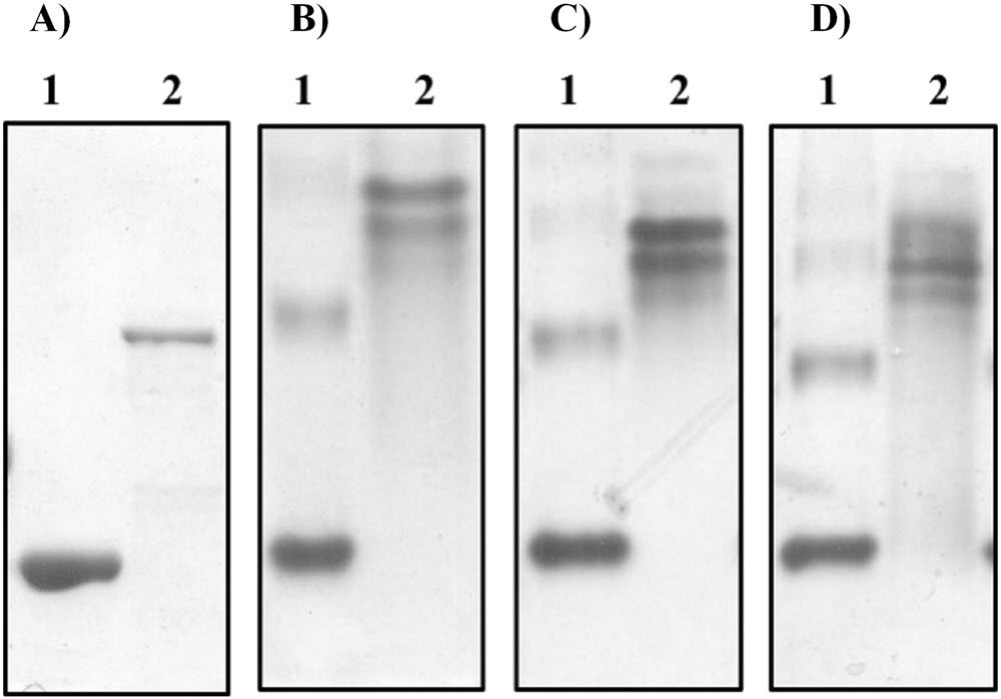
Binding of *Ec*Chi1 with the soluble polymeric substrates. Affinity non-denaturating electrophoresis was performed in 8% native PAGE gels by preparing with or without substrate. (**A**) Proteins (*Ec*Chi1 or non-interacting BSA) without substrate, while (**B**) with 0.1% (wt/vol) substrate glycol chitin, (**C**) CM-cellulose and (**D**) laminarin incorporated in gels. Proteins were visualized by Coomassie blue G-250 staining after electrophoresis. Lane 1. BSA, Lane 2. *Ec*Chi1.

Binding of *Ec*Chi1 to the soluble substrates was analysed using native PAGE with or without polysaccharides embedded in the gel. In presence of laminarin, CM-cellulose and glycol chitin there was a decrease in electrophoretic mobility of *Ec*Chi1 (Fig. 2). Among the three soluble substrates, the retardation in the mobility of *Ec*Chi1 was more by soluble glycol chitin than CM- cellulose, and laminarin suggesting specificity of *Ec*Chi1 towards chitinous substrates.

### Time-course of colloidal chitin and oligosaccharide degradation

**Figure 3:**
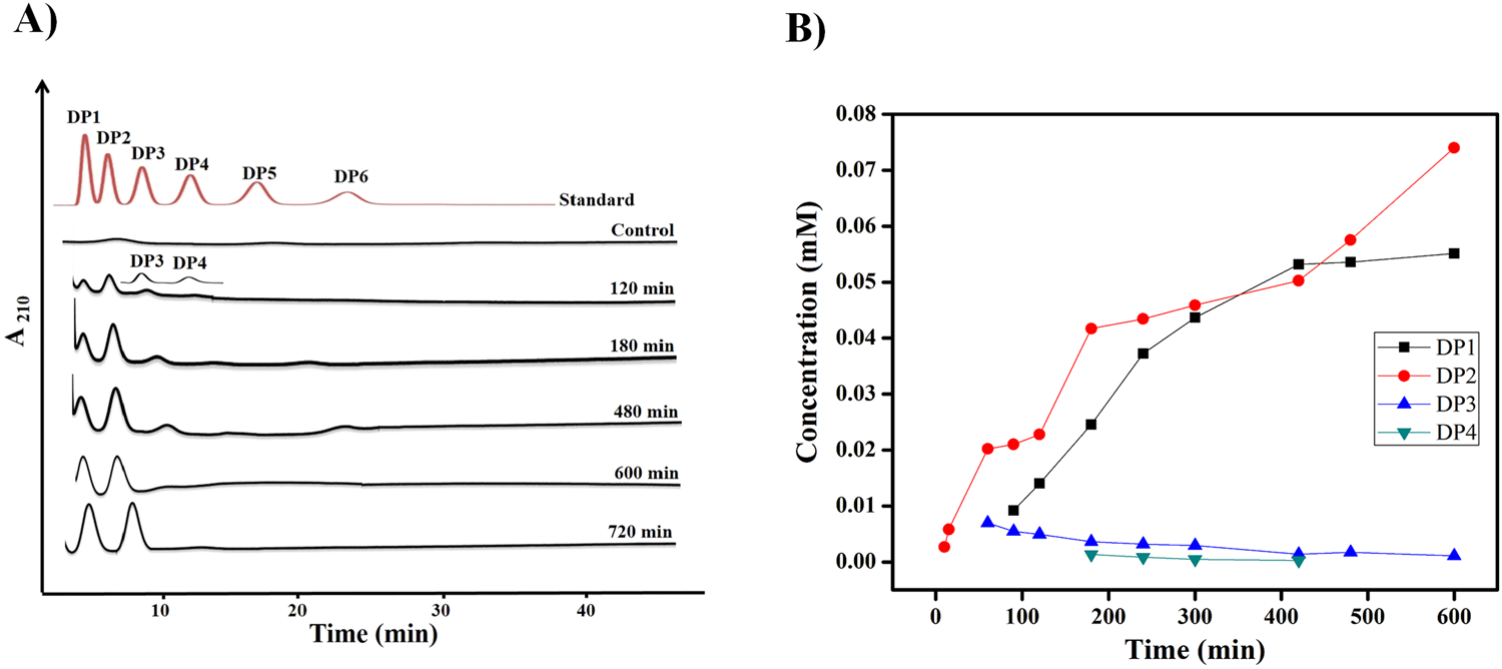
Hydrolysis of colloidal chitin by *Ec*Chi1. The *Ec*Chi1 (0.8 μM) was incubated with 30 mg/ml of the substrate in 900 μL reaction mixture for different time periods from 0 to 720 min at 40 °C. The reaction products were analyzed by binary gradient HPLC. (**A**) The topmost profile shows a standard mixture of CHOS ranging from DP1 to DP6. The remaining profiles are the reactions at different incubation times. Control represents the substrate without *Ec*Chi1 while standard represents CHOS ranging from DP1 to DP6. (**B**) Overview of the quantifiable CHOS products generated during hydrolysis. Products were quantified from respective peak areas using standard calibration curves of CHOS ranging from DP1 to DP4.

**Figure 4:**
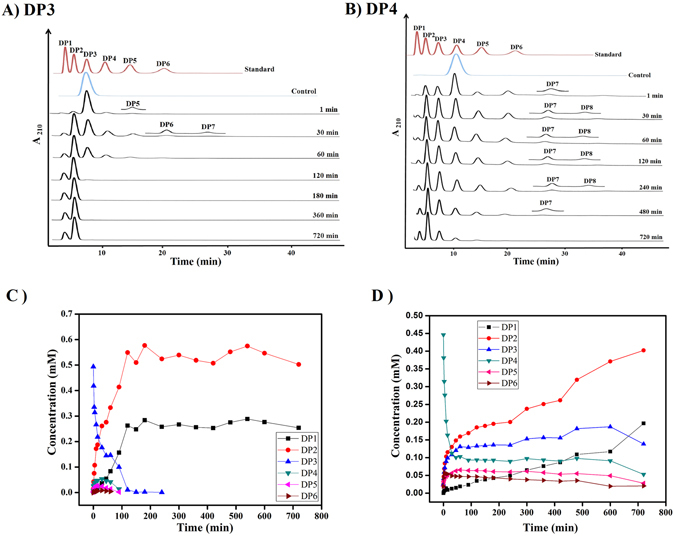
Time course hydrolysis and TG catalyzed by *Ec*Chi1 with DP3, DP4. The reaction mixture was incubated with 200 nM of *Ec*Chi1 and 1 mM of DP3/DP4 for different time periods from 0 to 720 min at 40 °C. Products were analyzed by binary gradient HPLC. (**A** and **B**) HPLC profiles of reaction products from DP3 (**A**) and DP4 (**B**) substrates. The topmost profile shows a standard mixture of CHOS ranging from DP1 to DP6 (**A** and **B**). The other profiles show the reaction products from DP3 and DP4 substrates at the indicated incubation times. The inset shows a magnified view of the low-peak-area products. Control represents the substrate without *Ec*Chi1. Overview of concentrations of CHOS products generated during reaction time courses with DP3 (**C**) and DP4 (**D**) substrates. Products were quantified from respective peak areas using standard calibration curves of CHOS ranging from DP1 to DP6.

**Figure 5:**
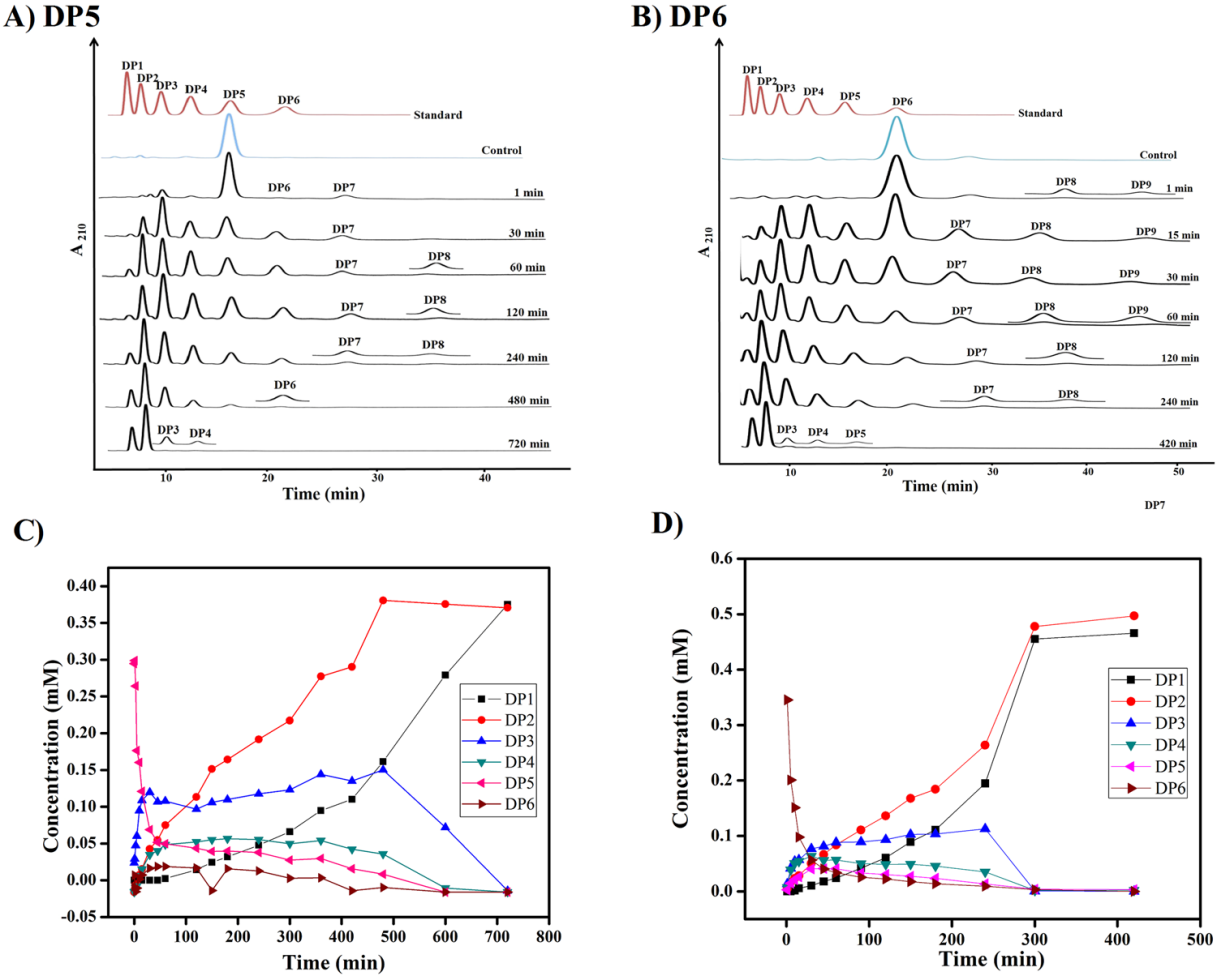
Time course hydrolysis and TG catalyzed by *Ec*Chi1 with DP5 and DP6. The reaction mixture products were analyzed by binary gradient HPLC. (**A** and **B**) HPLC profiles of reaction products from DP5 (**A**) and DP6 (**B**) substrates. The topmost profile shows a standard mixture of CHOS ranging from DP1 to DP6 (**A** and **B**). The other profiles show the reaction products from DP5 and DP6 substrates at the indicated incubation times. The inset shows a magnified view of the low-peak-area products. Control represents the substrate without *Ec*Chi1. (**C** and **D**) Overview of concentrations of CHOS products generated during reaction time courses with DP5 (**C**) and DP6 (**D**) substrates. Products were quantified from respective peak areas using standard calibration curves of CHOS ranging from DP1 to DP6.

*Ec*Chi1 released DP1 to DP4 hydrolytic products on polymeric colloidal chitin substrate with DP2 and DP1 as the major products (Fig. 3A and B). The concentration of DP1 increased with the extension of incubation time. *Ec*Chi1 hydrolyzed DP2 into DP1 after 240 min, and complete hydrolysis occurred by 420 min (Supplementary Fig. S3). Chitobiose was the major hydrolytic product from both DP3 and DP4 substrate (Fig. 4A and B). With DP5 substrate, a very little DP1 (1.4%) formed from 120 min. After prolonged incubation, DP1 was the dominant product with 37.5% followed by DP2 with 28% at 720 min (Fig. 5A and C). The hydrolysis of DP6 by *Ec*Chi1 was rapid with only 2.2% of DP6 at 120 min (Fig. 5B and D).

**Figure 6:**
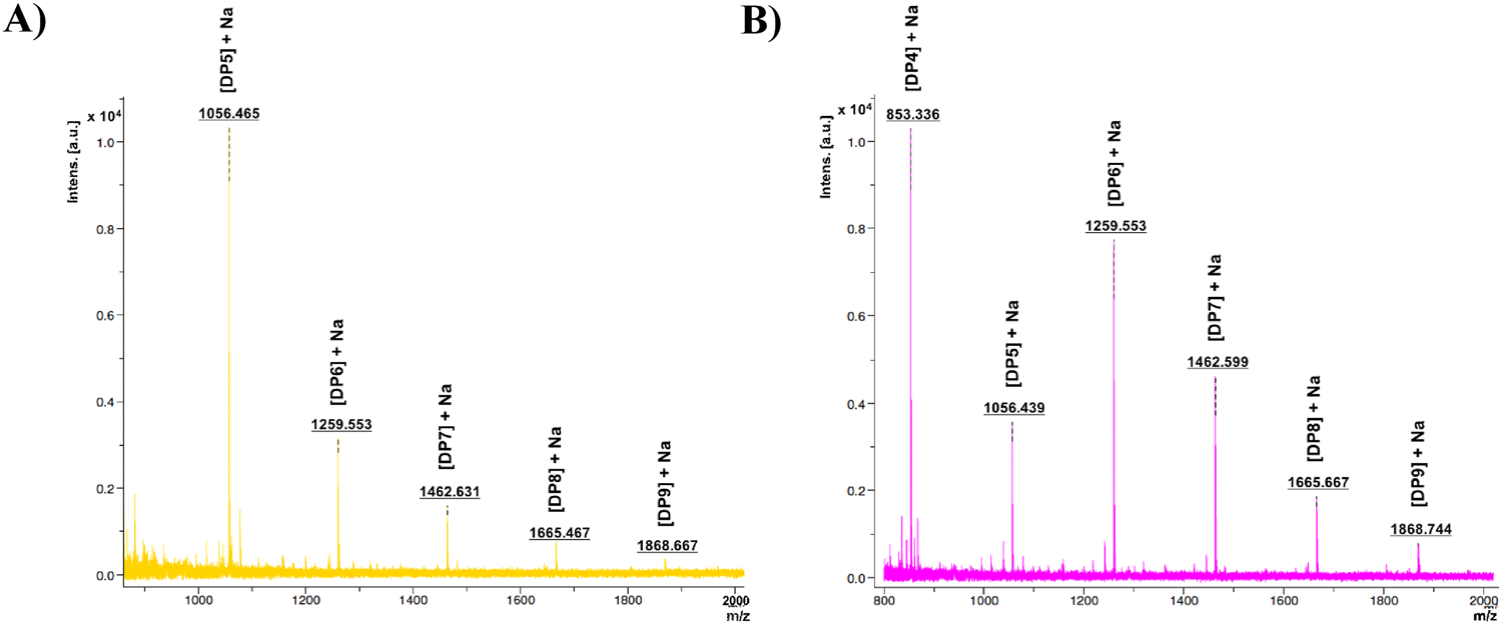
MALDI-TOF MS analysis of CHOS/TG products with DP5 and DP6 substrates catalyzed by *Ec*Chi1. The each 40 μL of reaction sample was concentrated under reduced pressure at 25 °C and dissolved in 10 μL of MilliQ H_2_O. The 2 μL of the reaction mixture was mixed with equal volume of 2,5-dihydroxybenzoic acid (2,5-DHB), and the resultant solution was subjected to mass measurements using an Ultraflex MALDI – TOF/TOF instrument. The masses shown here are with Na adducts.

*Ec*Chi1 exhibited high TG activity on chitotriose (DP3), chitotetraose (DP4), chitopentaose (DP5) and chitohexaose (DP6) substrates. With DP3 as starting substrate, TG products DP4-DP7 were detectable. DP4 and DP5 appeared from 1 min to 90 min, while DP6 and DP7 products appeared from 3 min to 60 min (Fig. 4A and C). Chitobiose was the major hydrolytic product from DP3 substrate. Use of DP4, DP5, and DP6 as starting substrates showed the formation of longer TG products (Figs 4B and 5A,B). With DP4 substrate, DP5-DP8 products formed due to TG, whereas DP9 was detectable as the longest TG product from DP5 and DP6 substrates (Figs 5B and 6). MALDI-TOF MS confirmed the products ≥DP6 of *Ec*Chi1. The products ranging from DP5 to DP9 and DP4 to DP9 were detectable with DP5 and DP6 substrates, respectively (Fig. 6A and B).

Reaction with DP4 substrate accumulated TG products from 1 min and lasted for 480 min, while DP8 product was detectable until 240 min. The TG products DP5 and DP6 reached a maximum quantity of 5.3% and 4.4% at 60 and 5 min, respectively (Fig. 4B and D). With DP5 substrate, DP6-DP9 products formed due to TG. DP6 formed from 1 min that reached to a maximum (2.5%) by 45 min, that was hydrolyzed to shorter CHOS (Fig. 5A and C). The CHOS DP7-DP9 were detectable TG products with DP6 substrate (Fig. 5B and D). The TG products formed from 1 min onwards and remained until 240 min.

The hydrolytic and TG activity of *Ec*Chi1 were assayed at low substrate and enzyme concentrations with DP4 substrate. *Ec*Chi1 exhibited remarkable TG activity even with the low substrate and enzyme concentrations. A 10-fold lower concentration of the substrate (100 μM) and enzyme (20 nM) resulted in the formation of DP5-DP7 as TG products until 30 min that was eventually degraded (Supplementary Fig. S4A). When the substrate concentration decreased by 20 fold (50 μM), the TG products, DP5 and DP6 formed between 0–8 min, which gradually decreased over time (Supplementary Fig. S4B). These results indicated that the TG activity of *Ec*Chi1 was proportional to the substrate concentration.

### Docking of *Ec*Chi1 with chitin tetramer

**Figure 7:**
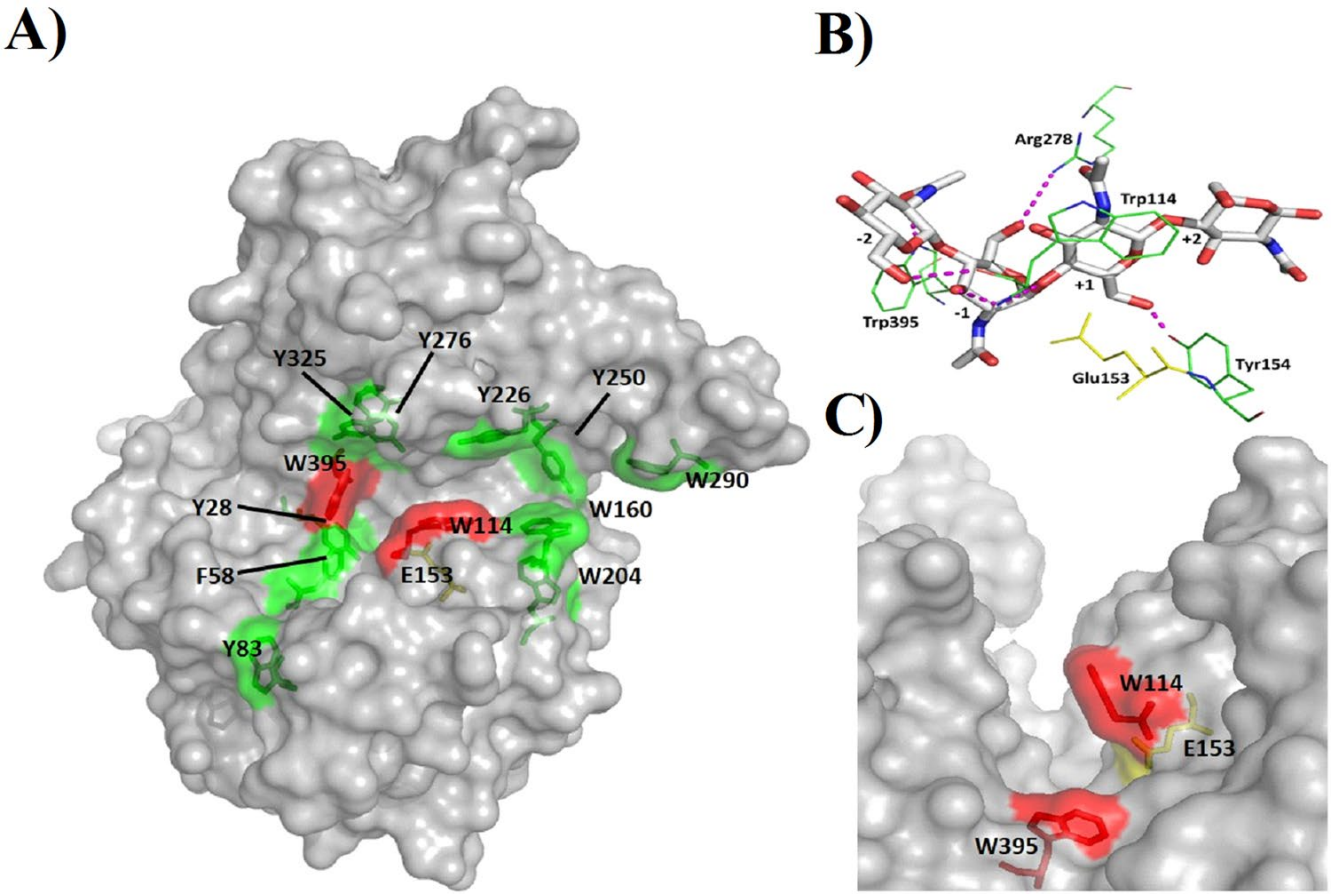
Substrate binding cleft of modelled *Ec*Chi1. (**A**) Aromatic residues lining along the substrate binding cleft (protein in surface representation and aminoacids in green and red sticks) and aminoacids interacting with chitin tetramer labeled in red colour. (**B**) Docked conformation of chitin tetramer (white sticks) and its possible interactions with residues (green lines) along with catalytic residue, Glu153 (yellow line) of *Ec*Chi1. (**C**) Deep active site cleft of *Ec*Chi1 showing catalytic (yellow colour) and aromatic (red colour) amino acids.

Sequence analysis revealed that *Ec*Chi1 is a single modular GH18 chitinase. The closest homologue of *Ec*Chi1 with a known crystal structure was chitinase II from *Klebsiella pneumoniae* (PDB: 3QOK; 86% sequence identity) which was used as the template to generate a 3D model. The stereo chemical quality of the generated model was checked for its quality through Ramachandran plot generated from PROCHECK analysis. The model showed 92.1% amino acids of modelled protein fell in the most favored region. Verify-3D graph also showed that 95.71% of the residues had an averaged 3D-1D score > = 0.2. Coordinates for chitin tetramer were extracted from the crystal structure of chitinase A from *Serratia marcescens* (PDB: 1NH6) and docked with modelled *Ec*Chi1 protein. *Ec*Chi1 complexed with DP4 substrate showed binding of DP4 from +2 to −2 subsites. Trp114 and Tyr154 and Arg278 and Trp395 are present at +2, +1 and −1, −2 positions, respectively. The residues forming interactions with ligand in the protein-ligand complex were Trp114, Tyr154, Arg278, and Trp395. Amino group in indole ring of Trp395 formed the hydrogen bond with a distance of 3.5 Å with the oxygen of acetamido group of non-reducing sugar moiety and a hydroxyl group on C6 of non-reducing sugar the formed hydrogen bond with 2.8 Å distance with the carboxylic group of Trp114, at −2 subsite (Fig. 7B). A terminal guanidium amino group of Arg278 and the amino group of Trp114 formed a hydrogen bond with 3.4 Å and 2.1 Å distances with the hydroxyl group of C6 and C4 of the penultimate non-reducing sugar moiety, respectively at −1 subsite. The hydroxyl group of Tyr154 formed a hydrogen bond with the hydroxyl group on C6 of sugar moiety at +1 subsite with 1.9 Å distance (Fig. 7B). *Ec*Chi1 has deep substrate binding cleft lined with aromatic residues that may play a major role in TG activity. The two aromatic amino acids Trp114 and Trp395 are located near to the catalytic center Glu153 (Fig. 7A and C).

## Discussion

The recent advances in whole genome sequencing technologies have paved the way to mine bacterial genomes for the genes and proteins of industrial importance. Search for new chitinases by utilizing the available genome information from diverse bacteria may provide chitinases with high TG activity for generating longer-chain CHOS. Phylogenetic analysis of chitinases from diverse bacterial members showed clustering of chitinases belonging to Enterobacteriaceae members along with a well characterized hyper-TG chitinase *Sp*ChiD with a few exceptions^17^. This observation suggested that mining Enterobacteriaceae members could be an option for novel TG enzyme. In the present study, we have identified and characterized a hyper-TG chitinase (*Ec*Chi1) from *Enterobacter cloacae*, a familiar representative species of family Enterobacteriaceae. Very few reports are available on chitinases and chitinolytic system of *Enterobacter* sp (Table 1). Among which, *Enterobacter* sp. G-1^18^, *E. aerogenes* ^19^, and *Enterobacter* sp. NRG4^20^ chitinases were characterized. ChiA was the first reported chitinase^21^ from an *Enterobacter* sp and shared 26% sequence homology with *Ec*Chi1. *Ec*Chi1 exhibited 87% sequence similarity with *E. aerogenes* chitinase, which did not have TG activity. There is no correlation between overall sequence similarity and TG as it depends on active site architecture of the enzyme and the orientation of water molecules at the catalytic centre which favors binding of incoming carbohydrate molecules, through strong interactions in the aglycon subsites^22^. The amino acid sequence of *Ec*Chi1 showed 87% identity to that of GH18 chitinase II (PDB ID: 3QOK) from *Klebsiella pneumoniae* for which no characterization was available. *Ec*Chi1 shared 75% identity with a hyper-TG chitinase of *S. proteamaculans* (*Sp*ChiD) (PDB ID: 4NZC)^11^. Nevertheless, many chitinases showing TG such as ChiA1 from *B. circulans* WI-12^23^, ChiA from *Vibrio harveyi* ^24^, and human chitotriosidase-1^25^ showed only 31%, 26% and 29% sequence similarity to *Ec*Chi1, respectively.

**Table 1:** Details of chitinases characterized from *Enterobacter* sp.

Strain name	Chitinase	Mol. Wt (kDa)	Optimum pH	Optimum Temp (°C)	TG activity	Reference
*Enterobacter agglomerans*	ChiA	61	ND	ND	Not reported	21
*Enterobacer* sp.G-1	N-acetyl glucosaminidase	92	6.0	45	Not reported	18
	ChiA	60	ND	ND	Not reported	61
*E nterobacer* sp. NRG4	ChiA	61	5.5	45	Not reported	20
*E. aerogenes*	Chitinase	42.5	6.0	55	Not reported	19
*E. cloacae* subsp*. cloacae*	*Ec*Chi1	42.5	5	40	Yes	Present study

ND – Not determined.

Enzymes show a pH range at which they are most active. *Ec*Chi1 was optimally active at slightly acidic pH (pH 5.0–6.0) similar to the chitinases A, B, and D from *S*. *proteamaculans* ^11, 26^, chitinase B from *S. marcescens* ^27^, *Paenibacillus sp*. D1^28^ and *Enterobacter* sp. NRG4^29^. *Ec*Chi1 optimally active at 40 °C comparable to *Sp*ChiB, *Sp*ChiC^26^, *Sp*ChiD^11^ and *Chitinibacter* sp. GC72^30^. The optimum temperature of *Ec*Chi1 was higher than many other chitinases such as *Vibrio* sp^31^ and *S. antarcticus* ^32^. However, some chitinases from *B. cereus* IO8^33^ and *Paenibacillus barengoltzii* ^34^ exhibited high optimal temperature than *Ec*Chi1.

Unlike pH and temperature optima, the kinetic parameters of *Ec*Chi1 were different from *Sp*ChiD. The K_m_ and catalytic efficiency (*k*_cat_/K_m_) of *Ec*Chi1 were 6 fold decrease and nearly 2 fold increase than *Sp*ChiD, respectively^11^. Among all the kinetic parameters, there is a significant correlation between K_m_ and TG, whereas no direct correlation between *k*_cat_ and TG activity was observed in chitinases^35^. The single mutants of ChiA-D313N and ChiB-D142N from *S. marcescens* showed 2 to 3 fold lower K_m_ and increased TG than the wild-type. The hydrophobicity of amino acids at substrate acceptor region and catalytic groove makes the enzyme efficiently bind to the substrates which resulted in less K_m_. The overall catalytic efficiency (*k*_cat_/K_m_) of *Ec*Chi1 was lower than ChiA, ChiB and, ChiC from *S. proteamaculans* ^26^ possible attributed to the lack of accessory domain (s) in *Ec*Chi1. The activity of *Ec*Chi1 on crystalline *α*-chitin and *β*-chitin was also low in comparison to colloidal chitin due to lack of accessory domains. The lining of aromatic amino acids in a substrate binding groove of *Ec*Chi1 (Fig. 7A) might responsible towards chitinous polymeric substrates. Similar findings were observed for *Sp*ChiD and *Stm*ChiA but not for *Stm*ChiB^11, 12^. *Ec*Chi1 had a very low activity on avicel (Fig. 1D). This could be due to the non-specific activity of *Ec*Chi1, as reported in a chitinase (ChiC) from *Pseudoalteromonas* sp. DL-6^36^. Various cations, particularly metal ions influence the activity of chitinases^37^. Certain ions such Cu^+2^ and Zn^+2^ act as either stimulators or inhibitors on chitinases^38^, not in the case of *Ec*Chi1.

The highly conserved catalytic motif (DxxDxDxE) and chitin-binding motif (SXGG) are positioned between 146–153 and 110–113 amino acids, respectively. Single amino acid residue present immediate to the chitin-binding motif and presence of α/β fold insertion plays a pivotal role in processivity^39^. The two aromatic amino acids Trp97 and Trp220 were crucial for processivity in ChiB from *S. marcescens* ^40^. Presumably, corresponding aromatic amino acids Trp114 and Tyr226 might tightly bind with the polymeric substrate and steer the processive action of *Ec*Chi1, implying that *Ec*Chi1 might be a processive enzyme (Fig. 7A and C). In addition, homology modelling studies of *Ec*Chi1 showed the presence of deep substrate binding cleft lined with twelve aromatic amino acids, and α/β fold insertion further support the characteristic feature of a processive enzyme. Analysis of product profile for dominance of even number oligosaccharides and the ratio of DP2/DP1 have been employed to determine an apparent degree of processivity^41, 42^. For example, the ratio of initial DP2/DP1 for two processive enzymes *Sm*ChiA and *Sm*ChiB had 30.1 ± 1.5 and 24.3 ± 2.0, respectively^42^. Upon degradation of colloidal chitin by *Ec*Chi1, DP1 was not detectable up to 60 min indicating a possible processive mode (Fig. 3B). At 90 min the DP2/DP1 ratio was 1.45, which further decreased with increase in time possible because of chitobiase activity of *Ec*Chi1.

HPLC analysis revealed that DP2 and DP1 are the major end products with colloidal chitin substrate similar to *Sp*ChiD and *Stm*ChiA^11, 12^. Gradual formation of DP1 from DP2 indicated chitobiase activity and DP2 was not a suitable substrate for TG. *Ec*Chi1 exhibited marginally lower *k*_cat_ when compared to *Sp*ChiD. The sequence analysis and modelling studies showed *Ec*Chi1 having a unique loop (Asn30 - Asp42) present at N- terminal region. The amino acids present at substrate binding cleft were occupied by the loop structure. The unique loop and aromatic residues highly conserved in single domain GH18 Enterobacteriaceae chitinases. The conserved amino acids Tyr28, Val35, and Thr36 might be responsible for chitobiase activity in *Ec*Chi1 similar to *Sp*ChiD^17^.

TG is a far-flung phenomenon in GH18 family chitinases, little known about the structural basis underlying this integral activity. *Ec*Chi1 showed significant TG activity on CHOS and produced long chain CHOS up to DP9. The product profile with DP3 substrate suggested that −1 to +2 or −2 to +1 subsites are possible for productive binding. DP3 was the minimum length of the substrate for *Ec*Chi1 to show TG activity. DP1-DP8 products were detected with DP4 substrate in which DP5-DP8 were TG products, whereas DP5-DP9 were TG products in *Sp*ChiD^11^. DP9 was the longest TG product detected with both DP5 and DP6 substrates. Similarly, incubation of 5 mM chitopentaose (DP5) with recombinant human chitotriosidase (HCT) resulted in the formation of the DP1-DP9^25^. No TG activity was observed by *Stm*ChiA with DP5 substrate^12^. *Ec*Chi1 synthesized long-chain CHOS even with low concentrations of substrate and enzyme. Amino acid residues positioned at acceptor binding site are assumed to be crucial for TG activity^43^. The known chitinases with TG activity were occupied with aromatic amino acids at +n subsites. Phe396&Trp275 and Trp220&Trp97 were occupied by the position at +2 and +1 subsites of ChiA and ChiB from *S. marcescens*, respectively^44, 45^. Similarly, Trp114, Tyr154 at +1, +2 subsites might help further in delivering efficient TG activity in *Ec*Chi1. In addition, Trp285 and Trp164 are occupying in +2 and +1 subsites of ChiA from *B. circulans* ^46^. It was further supported by a non-processive endochitinase from *S. marcescens*, *Sm*ChiC2 which lacks TG activity and aromatic amino acids at +1, +2 subsites^39^. Thus bulk aromatic amino acids present at positive subsites and the lining of aromatic amino acids at substrate binding cleft might be increased the binding affinities for upcoming sugar acceptor molecule thereby promoted TG activity in *Ec*Chi1.

In addition to processivity, single amino acid residue, present next to this chitin-binding motif (SXGG), plays an important role in TG^47, 48^. SXGG motif was extended with tryptophan in other well-known TG chitinases, *viz*., HCT^25^, *B. circulans* ChiA^46^, ChiA and ChiB from *S. marcescens* ^49^ and *Sp*ChiD in *S. proteamaculans* ^11^. In non-TG and non-processive endo-chitinases, such as *Sm*ChiC2 and *Ll*Chi18A, this tryptophan was substituted with small amino acid alanine indicated its probable role in TG^47, 50^. Trp114 was the corresponding amino acid in *Ec*Chi1 which may contribute to TG and processivity. In addition, the highly conserved novel loop was identified among the GH18 single catalytic domain chitinases from Enterobacteriaceae, showing their divergence from other groups^17^. This unique loop (Asn30 - Asp42) is present in *Ec*Chi1 may favor the interaction with the incoming substrate molecule.

The degree of endo-activity is best assessed as a probability of endo-mode initiation where concentrations of both insoluble reducing groups (endo-initiation) and reducing end groups (exo-initiation) upon substrate degradation are determined^51^. Hence, the observation of CHOS with DP higher than DP2 is indicative of *Ec*Chi1 having endo-activity as observed for *Sm*ChiC. Homology modelling based structural analysis also suggests that *Ec*Chi1 has structural features of an endo-acting enzyme with an apparent processive mode of action. *Ec*Chi1 was the first reported chitinase from *Enterobacter* sp with high TG. The capability to hydrolyze chitin with high TG activity suggests *Ec*Chi1 could be a suitable enzyme for industrial production of long chain CHOS, while it would also be of interest to know the possible physiological role of *Ec*Chi1.

## Methods

### Chemicals and enzymes

Genomic DNA of *E. cloacae subsp. cloacae* ATCC 13047 was used as a template to amplify the *Ec*Chi1 gene. The plasmid pET-28a (+) and *E. coli* Rosetta-gami II (DE3) (Novagen, Madison, USA) were used for heterologous expression. *E. coli* was grown in Luria bertani (LB) broth (1% peptone, 0.5% yeast extract, 1% NaCl) at 37 °C. The antibiotics kanamycin (50 µg ml^−1^) and chloramphenicol (25 µg ml^−1^) were added to the medium as required. Oligonucleotide primers were designed based on the DNA sequence available in the database and procured from Integrated DNA Technologies (Bangalore, India). Restriction enzymes, T4 DNA ligase, and Q5 DNA polymerase were from New England Biolabs (NEB). Isopropyl-*β*-D-thiogalactoside (IPTG), kanamycin and all other chemicals were purchased from Calbiochem, Merck (Darmstadt, Germany) or from Hi-Media Labs (Mumbai, India). The polymeric substrates α-chitin (extracted from shrimp shells), *β*-chitin (extracted from squid pen), and high molecular weight chitosan substrates of a fraction of acetylation (F_A_) = 0.10% (Mahtani Chitosan Pvt. Ltd.) and F_A_ = 25% (Sigma-Aldrich) were used for enzyme assay. Colloidal chitin and glycol chitin were prepared as described earlier^52^. CHOS with different DP were purchased from Seikagaku Corporation (Tokyo, Japan). Avicel was procured from Sigma-Aldrich (St. Louis, MO).

### DNA manipulations and cloning of *Ec*Chi1

*E. cloacae* genomic DNA (gDNA) was isolated as described by^53^. A gradient PCR, with appropriate gene-specific forward primer: 5′-CTC CCA TGG GAC TGA TGT CCG TG-3′and reverse primer: 5′-GGC CTC GAG CTT CGC TAA CTG GTT-3′ was used to determine the suitable annealing temperature for maximum amplicon yield. The amplicon was purified using PCR cleanup kit (MACHEREY- NAGEL GmbH & Co., Germany) and cloned into the *Nco* I and *Xho* I sites of the pET-28a (+). The construct was used to transform *E. coli* Rosetta-gami-II (DE3) for expression of the cloned gene. Transformants were selected on kanamycin and chloramphenicol containing LB plates. The nucleotide sequence of the insert was confirmed by automated DNA sequencing (Scigenom, Kerala, India).

### Expression and purification of *Ec*Chi1

Expression and purification of *Ec*Chi1 were done as described by ref. 52. The expressed protein was purified using Ni-nitrilotriacetic acid (Ni-NTA) affinity chromatography. Fractions of eluate having *Ec*Chi1were pooled and the protein purity was assessed by performing 12% SDS-PAGE. The purified *Ec*Chi1, in 50 mM sodium citrate buffer (pH 5.0), was stored at 4 °C for further characterization. The protein content of the sample was measured using a bicinchoninic acid (BCA) protein assay kit (Thermo Scientific, USA) where bovine serum albumin (BSA) as a standard.

### Dot blot assay

A dot blot assay was performed to detect the activity of purified *Ec*Chi1 as described by ref. 11. A composite gel mixed with 0.1% glycol chitin was prepared. Five micrograms of *Ec*Chi1 was spotted onto the gel and placed in the humid chamber at 37 °C overnight. After incubation, the gel was stained with 0.01% calcofluor white M2R in 0.5 M Tris-HCl (pH 8.9) for 10 min at 28 ± 2 °C. Finally, the brightener solution was removed. The gel was washed three times with distilled water for every 10 min at 28 ± 2 °C. The lytic zone was visualized on a UV transilluminator.

### Reducing end assay

Chitinase activity was measured by a reducing-end assay as described by ref. 54 with slight modifications. Recombinant *Ec*Chi1 was incubated with colloidal chitin in 50 mM sodium citrate pH 5.0 at 40 °C, 200 rpm for 1 h, followed by centrifugation at 13,600 × g, 4 °C for 30 min. A 40 μL of the clear supernatant containing reducing groups was mixed with 300 μL of the freshly prepared color reagent (0.5 M sodium carbonate, 0.05% potassium ferricyanide) and boiled for 15 min at 120 °C. Optical density was measured for 200 μL of each reaction mixture at 420 nm. One unit was defined as the amount of enzyme that liberated 1 μmol of reducing sugar per min. All the reactions were performed in triplicate.

### Optimum pH and temperature

To determine the optimum pH, the purified *Ec*Chi1, and colloidal chitin was incubated at 40 °C for 1 h in 50 mM buffers of different pH (2.0 to 10.0). Glycine-HCl buffer (pH 2.0), sodium citrate buffer (pH 3.0–6.0), sodium acetate buffer (pH 4.0–5.6), sodium phosphate buffer (pH 6.0–8.0), and glycine-NaOH buffer (pH 9.0 and 10.0) were used to determine optimum pH.

The effect of temperature on the activity of *Ec*Chi1 was assessed by incubating 3.5 μM enzyme with 30 mg ml^−1^ colloidal chitin in 50 mM citrate buffer, (pH 5.0) at 10, 20, 30, 40, 50, 60, 70 and 80 °C and measured chitinase activity.

### Steady-state kinetics

Kinetic parameters of *Ec*Chi1 were determined using colloidal chitin and chitobiose as the substrate. The reaction mixture (200 μL) containing 0 to 60 mg ml^−1^ of colloidal chitin and 0 to 1000 μM chitobiose substrate with *Ec*Chi1 in 50 mM buffer was incubated at 40 °C for 1 h with constant shaking at 200 rpm for chitinase assay. The unit of chitinase was defined as the release of 1 μmol of GlcNAc per second under standard experimental conditions. Specific activity was calculated in nkat/mg of protein. Kinetic values were obtained from triplicates of data fitting to the Michaelis-Menten equation *via* Non-linear regression using GraphPad Prism version 5.0 (GraphPad Software Inc, San Diego, CA).

### Substrate specificity

The substrate specificity of the purified *Ec*Chi1 was determined by replacing the colloidal chitin in the reaction mixture with different insoluble chitinous and non-chitinous substrates *viz*., *α*-chitin, *β*-chitin, colloidal chitin, glycol chitin (water-soluble chitin), chitosans (F_A_ = 0.1 and 25%), or Avicel (microcrystalline cellulose). *Ec*Chi1 (3.5 μM) was incubated with different substrates (2.5% wt/vol) at 40 °C for 1 h followed by reducing end assay. For each experiment, we individually calculated the reducing ends present in substrate alone and deducted the initial number of reducing groups from the value obtained from the enzyme-substrate reaction. One unit was defined as the amount of chitinase that liberated 1 μmol of reducing sugar per min.

### Soluble substrate binding

Soluble substrate binding was assessed as described by ref. 52. Different soluble polymeric substrates, *viz*., glycol chitin, laminarin, and CM-cellulose were incorporated in 8% polyacrylamide gels. BSA was loaded as a non-interacting protein. The electrophoresis was performed at 4 °C at a constant voltage of 80 V. The gels were stained with coomassie blue G-250 to detect the proteins. Binding was assessed visually or alternatively, the migration distances of the chitinases and protein marker were measured directly on the resolving gels.

### Effect of various additives

To determine the effect of various additives *viz*., metal ions (Ca^+2^, Cu^+2^, Fe^+2^, Mg^+2^, Mn^+2^, Hg^+2^, Zn^+2^), chelator (EDTA) and denaturants (Urea, SDS), of 1 mM each were added to *Ec*Chi1 with colloidal chitin as a substrate. After a pre-incubation for 1 h at 40 °C at 200 rpm activity was measured by reducing end assay. For each additive, the residual activity was calculated, considering the activity without additive was 100%.

### Time-course of colloidal chitin and oligosaccharide degradation

The hydrolytic activity of *Ec*Chi1 was assessed in a reaction mixture containing 1 mM substrate and 200 nM purified *Ec*Chi1 in 50 mM citrate buffer, (pH 5.0) at 40 °C over 720 min. The reaction was terminated by transferring 40 µL of the reaction mixture to an Eppendorf tube containing an equal amount of 70% acetonitrile and stored at −20 °C until analysis. Aliquots of 20 μL were injected into HPLC (Shimadzu, Tokyo, Japan) equipped with a ShodexAsahipack NH2P-504E column (4.6 mm [inner diameter] by 250 mm; Showa Denko K.K). Briefly, the mobile phase consisted of 70% acetonitrile and 30% MilliQ H_2_O, the flow rate was set to 0.7 ml min^−1^. The eluted CHOS were monitored at 210 nm. A CHOS mixture containing equal weights of oligomers ranging from DP1 to DP6 was used to generate standard chromatogram. Calibration curves were constructed separately for each CHOS in the mixture. The data points generated a linear curve for each CHOS with r^2^ values of 0.997 to 1.0.

### MALDI-TOF MS analysis

The products of the reaction mixture with DP5 and DP6 CHOS substrates were analyzed by matrix-assisted laser desorption ionization time of flight mass spectrometry (MALDI-TOF MS) after 45 min and 15 min, respectively. A 40 μL of the reaction mixture was concentrated and redissolved in 10 μL of HPLC-grade MilliQ H_2_O (Merck, Mumbai, India). Two microliters sample was mixed into the 2,5-dihydroxybenzoic acid (DHB) droplet and dried under a stream of air and analysis was done using Ultraflex MALDI-TOF/TOF (BrukerDaltonics GmbH, Bremen, Germany) with an autoflex 123 smart beam. The instrument was operated by the FlexControl 3.0 software package. All spectra were obtained using the reflectron mode with an acceleration voltage of 25 kV, a reflector voltage of 26, and pulsed ion extraction of 40 ns in the positive ion mode. The acquisition range was from m/z 800 to 2000. Peak lists were generated from the MS spectra using BrukerFlex Analysis software (version 3.0).

### Docking of *Ec*Chi1 with chitin tetramer

The protein sequence of *Ec*Chi1 was retrieved from NCBI Database (http://www.ncbi.nlm.nih.gov/) with accession number ADF62010.1 for modeling. To select appropriate templates for constructing 3D structure models of the *Ec*Chi1 protein, The BLAST program was used for sequence search against known 3D structure available in the Protein Databank (PDB) (http://www.rcsb.org/), The homology model program MODELLER v9.12^55^ was employed to generate 3D models of *Ec*Chi1 based on the crystal coordinates of chitinase II from *Klebsiella pneumoniae* (PDB: 3QOK). The stereochemical quality of the modeled protein structure was checked in Ramachandran plot^56^ using PROCHECK^57^. The compatibility of the model with its sequence was measured by Verify-3D graph^58, 59^. Chitin tetramer ligand was extracted from the 1NH6 crystal structure, using Discovery studio 4.0 and used for docking studies using Autodock 4.2^60^ via Auto Dock Tools (ADT) graphical user interface. Interactions with ligand were viewed in PyMol Molecular Graphics System 1.7.

## Electronic supplementary material

Transglycosylation by a chitinase from <i>Enterobacter cloacae</i> subsp. <i>cloacae</i> generates longer chitin oligosaccharides
