# Supplementary material for: Transglycosylation by a chitinase from Enterobacter cloacae subsp. cloacae generates longer chitin oligosaccharides
Source: Sci Rep. 2017 Jul 11;7:5113. doi: 10.1038/s41598-017-05140-3 (PMC5505975; doi:10.1038/s41598-017-05140-3)
Supplement: Supplementary file 1 — Transglycosylation by a chitinase from Enterobacter cloacae subsp. cloacae generates longer chitin oligosaccharides [file 41598_2017_5140_MOESM1_ESM.pdf]

SUPPLIMENTARY INFORMATION

Transglycosylation by a chitinase from *Enterobacter cloacae* subsp. *cloacae* generates longer chitin oligosaccharides

Mohan Krishna Mallakuntla, Papa Rao Vaikuntapu, Bhoopal Bhuvanachandra, Subha Narayan Das and Appa Rao Podile*

Department of Plant Sciences, School of Life Sciences, University of Hyderabad, Gachibowli, Hyderabad– 50046, Telangana, India.

Keywords:

*Ec*Chi1, Chitinase, Chitin oligosaccharides, Hydrolysis, Transglycosylation.

***Author for correspondence:**
Prof. Appa Rao Podile
Department of Plant Sciences
School of Life Sciences
University of Hyderabad
Gachibowli,
Hyderabad-500046, Telangana, India
Tel: +91-40-23134503
Fax: +91-40-23010120
E-mail: podilerao@uohyd.ac.in

Supplementary figure 1:

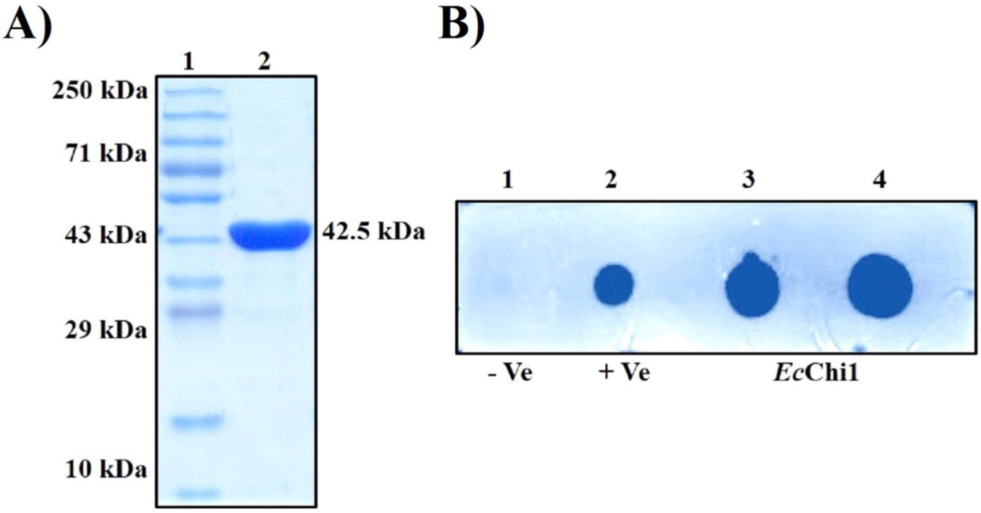

Fig. S1: Purification and activity analysis of *Ec*Chi1. (A) Recombinant *Ec*Chi1 (MW 42.5 kDa) was purified by Ni-NTA agarose affinity chromatography. Lane 1. Prestained protein marker, lane 2. Purified *Ec*Chi1. (B) Purified *Ec*Chi1 was spotted on glycol chitin substrate containing polyacrylamide gel and incubated overnight at 37°C in a humid chamber, and stained with Calcofluor white. The gel was placed on UV transilluminator to visualize lysis zone. Lane 1. Negative control (50 mM sodium citrate buffer pH 5.0), lane 2. Positive control (ChiA from *Enterobacter cloacae*), lane 3. 5 µg *Ec*Chi1, lane 4. 10 µg *Ec*Chi1.

Supplementary figure 2:

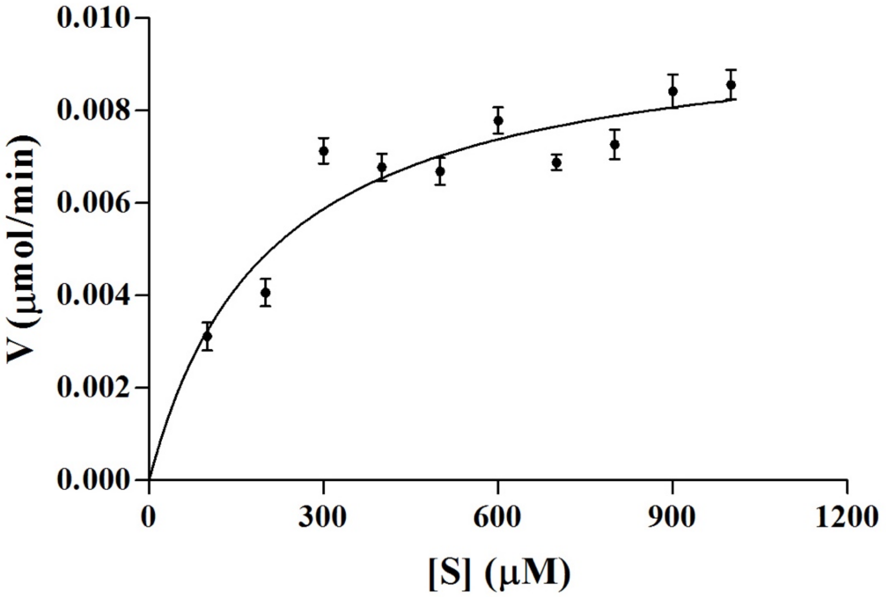

Fig. S2: Michaelis-Menten plot for chitobiose hydrolysis with *Ec*Chi1. Solid symbols are experimentally determined data for the rate of chitobiose hydrolysis versus substrate concentration. The solid line is the best nonlinear fit using the Michaelis-Menten equation. The error bars represent the standard deviations from three individual experiments.

Supplementary figure 3:

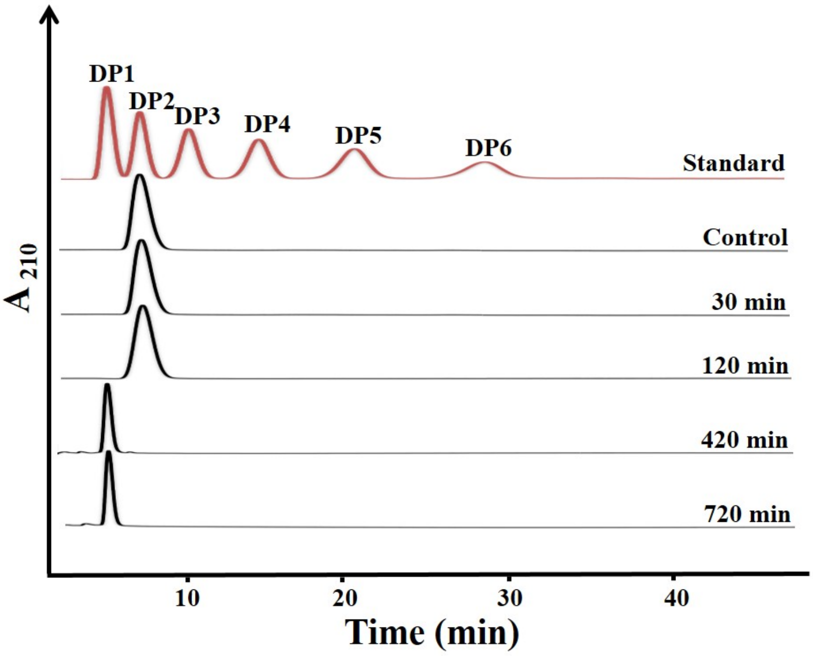

Fig. S3: Time course hydrolysis of *Ec*Chi1 with DP2 substrate. The reaction mixture products were analyzed by binary gradient HPLC. The topmost profile shows a standard mixture of CHOS ranging from DP1 to DP6. The other profiles show the reaction products at the indicated incubation times.

Supplementary figure 4:

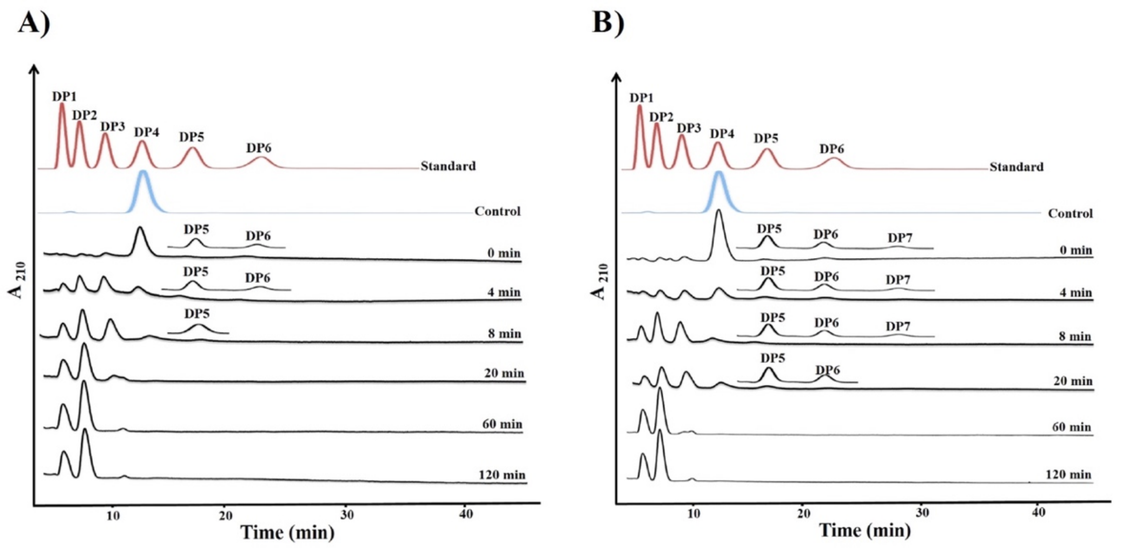

Fig. S4: Dose-responsive effects of *Ec*Chi1 with the low substrate (DP4) and enzyme concentrations on hydrolytic and TG activity. Fractions collected from reaction mixture were analyzed by binary gradient HPLC. (A) *Ec*Chi1 (20 nM) and DP4 substrate (100 µM); (B) *Ec*Chi1 (20 nM) and DP4 substrate (50 µM). The top most profile in each chromatogram shows a standard mixture of CHOS ranging from DP1-DP6. Insets show the magnified view of the low peak area products.
